# Detection of anticipatory postural adjustments prior to gait initiation using inertial wearable sensors

**DOI:** 10.1186/1743-0003-8-17

**Published:** 2011-04-06

**Authors:** Rigoberto Martinez-Mendez, Masaki Sekine, Toshiyo Tamura

**Affiliations:** 1 Graduate School of Engineering, Chiba University, Chiba, Japan

## Abstract

**Background:**

The present study was performed to evaluate and characterize the potential of accelerometers and angular velocity sensors to detect and assess anticipatory postural adjustments (APAs) generated by the first step at the beginning of the gait. This paper proposes an algorithm to automatically detect certain parameters of APAs using only inertial sensors.

**Methods:**

Ten young healthy subjects participated in this study. The subjects wore an inertial unit containing a triaxial accelerometer and a triaxial angular velocity sensor attached to the lower back and one footswitch on the dominant leg to detect the beginning of the step. The subjects were standing upright on a stabilometer to detect the center of pressure displacement (CoP) generated by the anticipatory adjustments. The subjects were asked to take a step forward at their own speed and stride length. The duration and amplitude of the APAs detected by the accelerometer and angular velocity sensors were measured and compared with the results obtained from the stabilometer. The different phases of gait initiation were identified and compared using inertial sensors.

**Results:**

The APAs were detected by all of the sensors. Angular velocity sensors proved to be adequate to detect the beginning of the step in a manner similar to the footswitch by using a simple algorithm, which is easy to implement in low computational power devices. The amplitude and duration of APAs detected using only inertial sensors were similar to those detected by the stabilometer. An automatic algorithm to detect APA duration using triaxial inertial sensors was proposed.

**Conclusions:**

These results suggest that the feasibility of accelerometers is improved through the use of angular velocity sensors, which can be used to automatically detect and evaluate APAs. The results presented can be used to develop portable sensors that may potentially be useful for monitoring patients in the home environment, thus encouraging the population to participate in more personalized healthcare.

## Background

Human equilibrium is inherently unstable unless a control system is continuously acting. The equilibrium system needs the coordination of three *subsystems*: sensory (vestibular organs, vision, cutaneous receptors, and proprioceptive sensors), skeletal (muscles, bones, tendons and ligaments) and central nervous system (brain and spinal cord). The central nervous system (CNS) counteracts equilibrium perturbations by mean of compensatory and anticipatory postural adjustments (APAs) [1-4]. While compensatory adjustments deal with actual perturbation of balance, the APAs precede perturbations [5]. The APAs consist of preprogrammed activation of the muscles, according to task parameters [6] and are important to minimize the effects of planned postural perturbations.

APAs are affected by the inclination of the floor [7], the magnitude of the forthcoming movement [8], posture [9], age [10,11], and several neurodegenerative diseases, such as Parkinson's disease [12], Huntington's chorea [13], and Down's syndrome. APAs are also affected in stroke patients and amputees [5]. The enormous importance of the APAs in the equilibrium system and its relationship with the CNS is an important reason to study APAs. In fact, some researchers have suggested the use of APAs as a method to evaluate the progress of patients with neurological disorders [13] or patients after a stroke [14] as well as to detect early clinical signs [15].

To date, APAs have been mainly detected and studied using electromyography (EMG), stabilometers, and motion-analysis systems. These systems have been proven effective; however, the cost and complexity of taking measurements and performing subsequent evaluations have limited their use to hospitals and well-equipped laboratories.

In an effort to avoid these limitations, some researchers have proposed the use of low-cost inertial sensors as an alternative to evaluate human movements for example normal gait [16-19], sway [20], to detect falls in elderly people [21], to detect changes in posture [22] and also to measure APAs [23,24,15].

The results of those studies have already demonstrated the capability of inertial sensors to detect and evaluate APAs prior to step but, until now, only accelerometers have been used. Moreover, only the results of two-dimensional measurements of APAs, i.e., anteroposterior (AP) and mediolateral (ML), have been presented.

With current advances in microelectronics and micro-electromechanical systems (MEMS), it is easy to find small and inexpensive devices that are capable of measuring not only acceleration, but also angular velocity in three spatial dimensions. The use of triaxial sensors, accelerometers, and angular velocity could improve the detection and evaluation of the APAs.

Furthermore, algorithms presented in previous papers depended on other systems such as stabilometers or video markers to detect the end of the APAs, which downgrade the use of inertial sensors as a tool to detect the APAs.

The availability of inertial sensors and the potential to use them as a standalone system to measure APAs makes it possible to design systems that enable the population to more frequently participate in the prevention and early prediction of diseases [25].

Due to the reasons presented above, the authors believe it is necessary to develop both a method to characterize inertial sensors signals in three dimensions and an algorithm to detect APAs reliably.

The aim of this study was to characterize the detection of APAs prior to gait initiation using a triaxial inertial wearable sensor attached on the lower back. The typical APA waveforms in young healthy subjects are presented. Additionally, a simple algorithm to detect the beginning and end of APAs using only the inertial sensors is proposed. The algorithm used is simple enough to be implemented in low computational power devices such as microcontrollers or digital signal processors (DSP) to achieve a wearable device capable of functioning without the need for a computer. The end of APAs calculated using this algorithm was compared with those values detected using a footswitch, device that determine the beginning of the step more accurately. By definition, the beginning of the step is the end of the APAs.

## Methods

### Subjects

Ten subjects (7 men, 3 women) with no previous history of neurological disorders or equilibrium problems participated in this study. Their average age was 26 ± 3 years (average ± SD), height was 165 ± 8 cm, and weight was 60 ± 10 kg. Subjects with corrected vision wore their glasses during the study. All subjects were right-handed.

Young healthy subjects were preferentially used because the main purpose of this study was to evaluate and characterize the use of inertial sensors for detecting APAs and to compare the results with those obtained employing typical methods. In addition, the protocol involves several trials for each subject. An elderly person or patient might not be able to perform these repetitions. Before the test, the subjects were informed of the purposes and conditions of the test and were asked to sign a consent form developed by the ethics committee of Chiba University. The study conformed to the standards set by the Declaration of Helsinki.

### Equipment

A stabilometer (ANIMA G-620; Anima Inc., Tokyo, Japan) was used to measure the center of pressure displacement (CoP). A footswitch consisting of two square plates (3 × 3 cm) was composed of a conductor and moldable material separated by a soft non-conductor material. A special shape of the non-conductor material was used to allow electrical connection when the mass over the sensor was higher than 4 kg with an acceleration of 1 g. The use of a moldable material prevents any discomfort to the subjects that may affect normal posture and stepping.

Two inertial wearable sensors units, each containing two type of sensors in the same unit, a triaxial acceleration sensor (MMA72260Q; Freescale, Austin, TX), and a triaxial angular velocity sensor composed of two ENC-03RC sensors (Murata, Tokyo, Japan) and one X3500 sensor (Epson, Tokyo, Japan), both, the ENC-03RC and the X3500 are angular velocity sensors, mounted orthogonally. The inertial sensor units were fully developed in our laboratory, and the electronic design and characteristics of the sensors permit a measuring range of ± 1.5g, a sensitivity of 800 mV/g, and a range of response frequency from 0Hz to 28 Hz for the accelerometer. For the angular velocity sensors, we achieve a sensitivity of 16.8 mV/deg/s with a range of ± 80 deg/s and a response frequency of 0.01-28 Hz. The analogue signals of all sensors were converted into digital using a 12-bit ADC built into a digital signal controller (DSC), dsPIC30F3012 (Microchip, Chandler, Arizona, USA). The signals were sampled at 100 Hz. The DSC sends the signals to a computer via Bluetooth using a ZEAL-S01 module (ADC technology, Inc., Tokyo, Japan). The data were received in a computer using an *ad hoc *program made with Visual Basic 2005 (Microsoft, Redmond, WA). The data transmitted included an algorithm for detection of data losses, which ensures the reliability of data transmission.

The resulting inertial sensor units also provide three more inputs to connect extra analogue sensors, if required. The size of each unit is 93 mm length, 64 mm width, and 20 mm height, with a weight of only 110 g including the battery.

Each unit can run for more than 4 hours using a rechargeable 9 V battery, 250 mAh. The accelerometers were calibrated by measuring their outputs under controlled inclination. For example, at 0°, 90°, and 180°, the values were 1 g, 0 g, and -1 g, respectively. The resolution obtained with these sensors and electronic design was 0.001 g/bit for the accelerometers and 0.047 deg/s/bit for the angular velocity sensors. The RMS noise was lower than 0.005 g for the accelerometers and lower than 0.12 deg/s for angular velocity.

Wearable sensors were chosen because they have a small size and mass. Furthermore, the absence of wires enables us to obtain measurements with minimal disruption of the natural movement of the subjects.

### Placement of sensors

**Figure 1:**
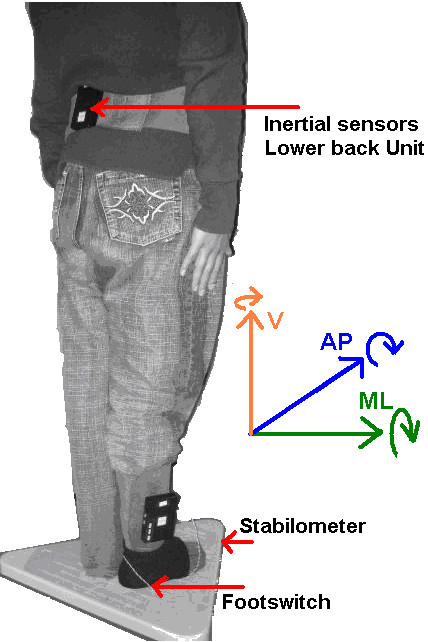
**Placement of sensors**. ML stands for mediolateral, AP stands for anteroposterior and V stands for vertical axis. The straight arrows indicate positive values of acceleration and the curved arrows indicate positive values of angular velocity.

One unit was attached to the lower back, around the L3-L4 vertebra. This position was chosen due to the proximity to the center of mass (CoM) of the human body. A second inertial sensor unit was attached to the lateral side of the ankle of the dominant leg. A footswitch was connected to this unit enabling the detection of the beginning of the step and allowing the recorded signals to be synchronized with the sensor on the trunk. The footswitch, which was attached to the heel of the dominant leg around the area of the calcaneus bone, was attached to the skin using an elastic sock to avoid movement, see Figure 1. The comfort of the subjects was assured by checking with each participant before the beginning of the experiment.

The stabilometer was placed on a plain surface; the area in front of the stabilometer was prepared to have the same level as the stabilometer to allow a natural step on flat surfaces. The stabilometer provided a synchronization signal, which was connected to one of the analog inputs of the inertial unit attached on the ankle.

### Test procedure

The subjects stood on the stabilometer upright and barefoot in a comfortable position with their arms at their sides. The foot position for each trial was controlled by asking the subjects to put their feet on the marks drawn on the stabilometer. The separation between the feet was of 2 cm and their longitudinal axis parallel to the anteroposterior (AP) plane.

At the beginning of the experiment, the subject focused on a mark at eye level on the wall at a distance of 3 m. Subjects were asked to take a step forward on hearing a command from the experimenter. The time for the command was chosen randomly without previous announcement. Before recording, the subjects were asked to perform a step to confirm whether they had comprehended the instructions correctly. Each subject performed five trials, and each trial lasted for 10 s, starting when the subject was standing on the stabilometer and finishing after subject completed a step. This trial duration was found to be long enough to allow for minimization of the natural sway before the step. After the step, the subject waited until the 10 s recording was finished and then went back to the initial position for the next trial. There was a 1-minute gap between trials, which was enough to prepare the equipment for the next recording. Studies have reported no changes in APA patterns as a result of fatigue [26]; therefore, a rest period was considered unnecessary.

### Data preparation and analysis

Signals were analyzed offline using MATLAB^^® ^^(MathWorks, Natick, MA). The signals for each trial were cut 3 s before the step and 2 s after the point indicated by the onset of the footswitch. Regarding to inertial signals, only data from the sensor attached to the lower back were analyzed, the sensor unit on the ankle was used mainly to read the footswitch and stabilometer synchronization signals rather than measure the inertial signals on the ankle thus, the inertial data of the sensor unit on the ankle was not considered. Bias for all signals was eliminated by simple subtraction of the average signal evaluated during the first 2000 ms. The data from the accelerometers were low-pass filtered using a second-order zero phase Butterworth filter. This elimination of bias minimizes the possible effects of the tilt in the accelerometers. The standard deviation (SD) of the baseline of each signal during the first 2000 ms was calculated. This signal was multiplied by a factor (F) and used as a threshold to detect the onset and end of the APAs In order to find the best results, several cutt-off frequencies were tested for the filter (2.5 Hz, 3 Hz, 3.5 Hz and 5 Hz) and several F values were used for the threshold calculation (F = 2, 3, 4, 5 and 6)

**Figure 2:**
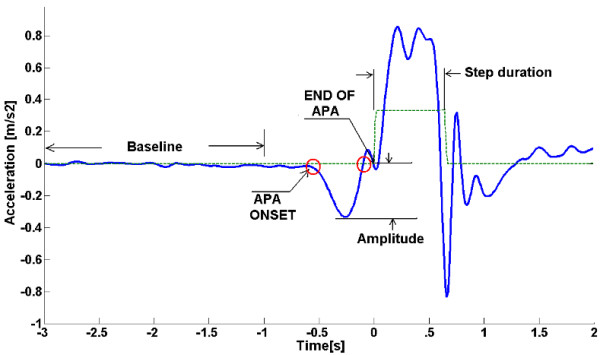
**Method for detection of APA**. Method for the detection of APA onset for an acceleration signal in the ML axis. All the signals were low-pass filtered. The standard deviation of the baseline during the first 2 s was multiplied by a factor (F) and used as a threshold (Th). The onset of the APA was determined by the time when the signal was >Th. The end of the APA was determined by the time when the signal returned to the baseline level. The foot switch (dotted line) was used to measure the time between the beginning of the step and the end of the APA. The same method was applied for all the signals.

The duration of the APAs was determined as the time between the onset of the signal of interest and the onset of the footswitch sensor (end of the APA). The Figure 2 exemplifies this method of detection using a mediolateral signal.

The duration and amplitude of the APA were measured for each sensor and each trial.

### Statistics

The internal consistency reliability of the APAs was evaluated using an average inter-item correlation test. The first step was to check whether the signals detected by the inertial sensors showed consistency among trials, both within and between subjects. The signals recorded by each sensor for all the trials for the same subject were compared. The inter-item correlation coefficient was calculated for each sensor and for all signals for each subject. This correlation served as an indicator of the degree to which the subject repeated the same APAs between trials. The repeatability of the data for each individual was also evaluated. The Pearson's r value was calculated to assess the correlation between the duration calculated using different sensors.

## Results

### Repeatability of APAs and waveforms

First, it is necessary to determine whether the APAs measured by the inertial sensors had internal consistency, i.e., if the waveform was consistent between trials for the same subject. An average inter-item correlation test was used for this purpose. The test calculates the correlation between each pair of signals and then calculates the average of all these resultant correlations. Table 1 shows the values calculated for each signal from the inertial sensors, as well as the signals from the stabilometer.

**Table 1:** Inter-item correlation results for all the signals subject by subject

Subject	Acceleration			Angular velocity			CoP displacement	
	ML	AP	UD	ML	AP	UD	ML	AP
**1**	0.88	0.87	0.83	0.69	0.56	0.64	0.99	0.99
**2**	0.87	0.92	0.72	0.80	0.80	0.73	0.99	0.98
**3**	0.82	0.93	0.65	0.83	0.60	0.77	0.97	0.95
**4**	0.84	0.67	0.50	0.69	0.55	0.77	0.97	0.98
**5**	0.83	0.83	0.75	0.88	0.81	0.94	0.98	0.99
**6**	0.51	0.97	0.78	0.83	0.54	0.65	0.98	0.98
**7**	0.87	0.84	0.83	0.84	0.74	0.77	0.93	0.99
**8**	0.93	0.97	0.90	0.93	0.89	0.88	0.99	0.99
**9**	0.80	0.92	0.83	0.93	0.83	0.68	0.98	0.86
**10**	0.57	0.84	0.72	0.84	0.70	0.69	0.98	0.93

**average**	**0.79**	**0.88**	**0.75**	**0.83**	**0.70**	**0.75**	**0.98**	**0.97**

***SD***	*0.14*	*0.09*	*0.11*	*0.08*	*0.13*	*0.10*	*0.02*	*0.04*

Inter-item correlation results for all signals from all the subjects (*p *< 0.05) in the worst case scenario. All signals were low-pass filtered at 3 Hz and evaluated over 5 s. A higher value means that the pattern of movement is very similar between trials; a lower value indicates that the movement is different between trials. Ideally, the value should be 1.

An inter-item correlation value close to close to 1.0 indicates that the signals are very similar between trials, i.e., the subject repeats the same movement.

**Figure 3:**
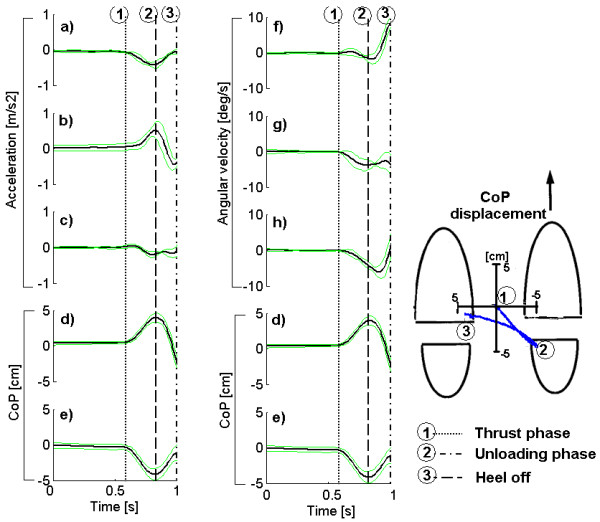
**Example of signals from the inertial sensors and stabilometer for one subject**. Example of signals from the inertial sensors and stabilometer for one subject 1 s before the step. a) acceleration in the ML plane, b) acceleration in the AP plane, c) acceleration in the vertical plane; f) angular velocity in the ML plane, g) angular velocity in the AP plane, h) angular velocity in the vertical plane. d) is the CoP displacement in the ML plane, and e) is the CoP in the AP plane. Note that the CoP signal is repeated to allow for an easier comparison of the main APAs characteristics with the inertial sensors. The dotted lines show the beginning of each phase of the APAs. The green lines indicate the SD of the signals for 5 trials.

At the bottom of Table, 1 the inter-item correlation values are averaged to provide an idea of which signals are more consistent. It is noteworthy that the stabilometer signals (CoP) have better internal consistency and reliability than do the inertial sensors; however, the inertial sensors still have a maximum consistence of 88% (acceleration-AP) and a minimum of 70% (Angular velocity-AP). Figure 3 shows the averaged waveforms for one subject using the five trials.

It is also interesting to calculate the similarity of signals among subjects. Specifically, how similar is the movement among different subjects? To answer this question the same inter-item correlation test can be applied. This time, the averaged signals over five trials for each subject were used. The values are shown in Table 2.

**Table 2:** Inter-item correlation values for each signal among subjects

*Sensor*	*Acceleration*			*Angular velocity*			*CoP displacement*	
**Axis**	**ML**	**AP**	**UD**	**ML**	**AP**	**UD**	**ML**	**AP**

**r (*p *< 0.05)**	0.55	0.44	0.55	0.13	0.2	0.56	0.6	0.63

Inter-item correlation values for each signal among subjects. These values indicate the similarity in patterns among several subjects.

The results indicate that the angular velocity in the ML and AP planes shows the greatest difference among subjects. These results suggest that the tilt of the trunk is different for each subject even when the CoP and acceleration in the ML plane show similar patterns.

Our results show a "mirror effect" between CoP displacement and acceleration signals. While the CoP movement is rearward toward the stepping foot, the acceleration is opposite, i.e., forward and toward the standing foot. This effect has already been published and explained in [1] and was found in previous studies using accelerometers. These results demonstrate the consistency of our method in detecting APAs.

### Duration of APAs

In previous studies measuring APAs using accelerometers, the beginning of the APA was determined by the time at which the amplitude of the signal exceeded a threshold. The threshold was given by the standard deviation (SD) of the signal when the subject was standing still multiplied by a factor of two [15]. In other studies, it was determined visually by detecting the first change of the signals using the CoP displacement [24]. In other cases, the end of the APAs was detected by using video systems (cameras and markers), [23] determining the end of the APA when the foot was raised; or using the CoP displacement [15] by defining the end of the APA as the time when the CoP in both ML and AP planes returned to baseline (below the threshold). In all cases, the beginning of the APA was determined using a system different from the inertial sensors; the use of inertial sensors does not make much sense if reference to other, more complex devices determines the end of the APAs.

In the present study, we used the same methods to detect the beginning of the APAs, but the end was determined using only the inertial sensor signals.

The threshold method was used to detect the beginning and the end of the APA. Values for the onset were determined by the time when the signal surpasses the threshold, and for the end, when the signal returns to baseline or is lower than the threshold. As explained in Figure 2, the threshold is calculated using the SD of the baseline during the first 2 s of standing, multiplied a factor (F).

The end of the APA was calculated for each signal and each combination of factors and cut-off frequencies. The calculated time was compared with the time determined by the footswitch, which was considered as the true end of the APA for this study.

**Figure 4:**
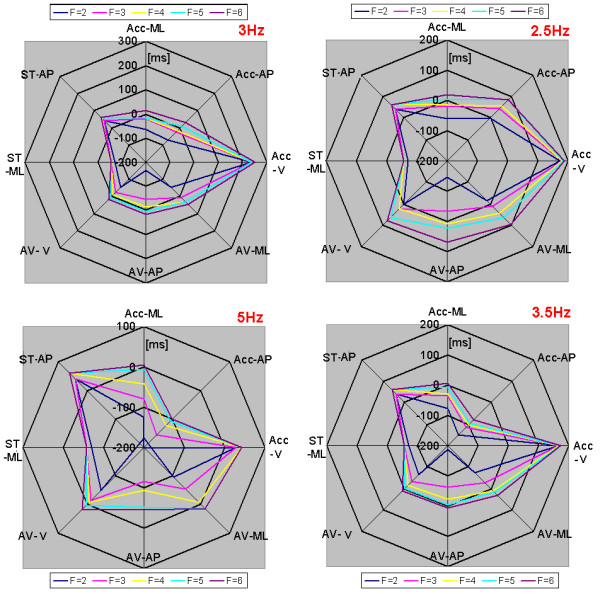
**Determination of the end of the APA using several threshold levels and cutt-off frequencies**. Detection of the end of APAs using several threshold levels (TH) and cut-off frequencies. The threshold is determined by the baseline of the signal multiplied by a factor (F). "Acc" stands for acceleration signals, "AV" stands for angular velocity sensors, and "ST" stands for stabilometer, which measures CoP displacement. The values are in milliseconds [ms]. A negative value denotes a time before the footswitch detected the leg being raised, and positive values denote a time after the footswitch detected that the leg was raised. Ideally the values should be either zero or negative values close to zero, never positive.

The results of this test are shown in Figure 4. Ideally, the lines in the graphs should be zero or very close to zero, which would indicate that the algorithm is detecting the end of the APA in exactly the same manner as the footswitch. The graphs show that the CoP-AP always identified the end of the APA after the true end, defined by the footswitch. In contrast, the CoP-ML identified the end before the true end occurred.

The cut-off frequencies that showed the least amount of dependence on the factor value were 3 Hz and 3.5 Hz, and the factor values that were closer to zero were 4, 5, and 6. The best-fit signals for detection of the end of APA were those from the angular velocity sensors, specifically the angular velocity in the anteroposterior plane (AV-AP) and the angular velocity in the vertical plane (AV-V).

**Figure 5:**
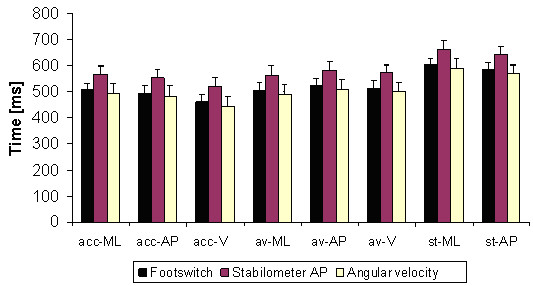
**Duration of APAs calculated using three different signals to determine the end f the APA**. Duration of APAs calculated using the footswitch, stabilometer (CoP-AP), and the angular velocity sensor (UD plane) used to determine the end of the APAs. The actual end of the APAs is considered to be determined by the footswitch.

Figure 5 shows the average duration of the APAs calculated using the footswitch, the CoP-AP, and the angular velocity sensor AP signals to determine the end of the APAs. The duration calculated using the CoP-AP is 10% larger than the real duration of the APAs, i.e., that detected using the footswitch. The duration using the angular velocity AP is 2.8% shorter than the actual duration. This result suggests that angular velocity in the vertical plane (V) instead of the accelerations signals should be used to detect the end of the APAs using a factor (F) equal to or greater than 4.

**Figure 6:**
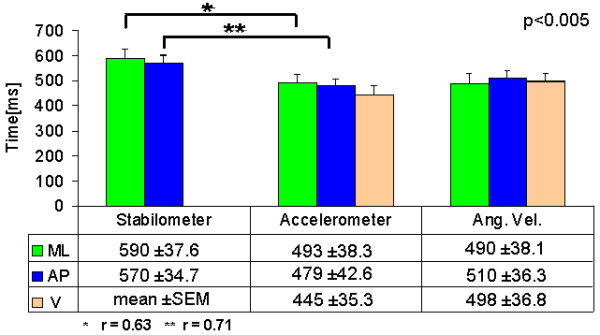
**Average APA duration for each sensor using the angular velocity in the vertical plane (AV-UD) to detect the end of the APA**. The beginning of the APA was considered to be when the each signal was greater than 3 times the standard deviation of the baseline during the first 2000 ms of each signal, and the end was determined by the time when the signal went back to baseline level. All signals went through a LPF zero phase (Butterworth Fc = 3.5 Hz).

Figure 6 shows the results of the average APAs duration detected by each sensor using a cutoff frequency of 3 Hz and a factor (F = 4). The end of the APA was determined by the angular velocity in the AP plane (AV-V), the correlations between CoP and accelerometer detected durations were (*r = 0.63) in the ML plane and (**r = 71) in the AP plane, *p *< 0.05.

The duration of the APA was detected with all sensors; the accelerometers show a lower APA duration, although the trends are the same as that of the CoP, the duration in the ML plane is longer than the duration in the AP plane.

### Amplitude of APAs

**Figure 7:**
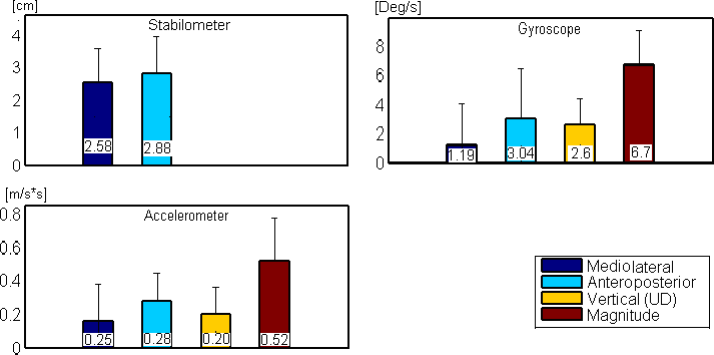
**Average amplitude values for all sensors**. The results show similar trends; the amplitude in the ML is lower than that in the AP plane. The magnitude of acceleration and angular velocity was calculated using the Euclidean norm.

The maximum amplitudes of the APAs for each type of sensor were measured, and the results are shown in Figure 7. The overall results suggested that both types of inertial sensor had similar behavior to the stabilometer with respect to the APA amplitude. The amplitude detected for the ML axis was smaller than that detected in the AP axis. These results suggest that the inertial sensors are able to detect changes in amplitude similar to the stabilometer.

## Discussion

### Repeatability of APAs

The results comparing the APA waveforms within subjects showed high correlation coefficients, indicating similarity within subjects repeating the same movement. The angular velocity in the AP plane (AV-AP) had the lowest correlation value. These results suggest that the tilt of the trunk in the AP plane was slightly different between trials. This result may be due to different patterns of arm movements or, more likely, different activation patterns of the abdominal muscles. None of the inertial sensors reached values similar to the CoP, which could due to the SNR of the inertial sensors. However, the authors consider that the values achieved are good enough to be considered repetitive.

Intersubject repeatability for the inertial sensors also shows similar values to that achieved by the stabilometer, which confirms the viability of measuring the APAs with inertial sensors. The angular velocity sensor in the AP plane showed the lowest correlation coefficients, indicating that these signals were markedly different between subjects (Table 2) and suggesting a different tilt response within the same subject. A study using EMG might be useful to determine the reason of that difference.

### Duration of the APAs

The duration of the APAs was determined using a simple algorithm based on a threshold level. The angular velocity signal in the vertical plane (AV-V) was best able to detect the end of the APA using this simple algorithm. It was demonstrated that the end of the APAs detected using the CoP was delayed by approximately 50 ms with respect to the true end of the APAs. This fact was not considered in previous studies that used this signal to determine the end of the APAs [15].

The use of angular velocity sensors, specifically in the vertical plane (AV-V) and anteroposterior plane (AV-AP), to detect the end of the APAs is recommended instead of the use of acceleration signals.

### Waveform and amplitude

As expected, the waveforms resulting from the APA movements were similar for the inertial sensors and the stabilometer, especially in the AP and ML planes. However, the peaks of these APAs were inverted in relation to the CoP. While acceleration describes a forward movement, the CoP has a rearward movement component. These results were similar to those reported previously [23,24,15].

The amplitude of the APA is another important factor, as demonstrated by Mancini [15] in a study measuring the APAs of Parkinson's disease patients. The patients showed lower APA amplitude than did healthy control subjects. Our results indicated a lower amplitude in the ML plane compared with the AP plane for all sensors. Our results are consistent with those presented in [27], but not [15]. This could be due to the restriction on the initial stance and the separation between feet used in [15].

## Conclusions

In this study, we examined the capabilities of inertial sensors (angular velocity and accelerometers) for detecting and evaluating various characteristics of anticipatory postural adjustments (APAs). We calculated the amplitude and duration of APAs while varying signal filtering and the threshold for detection of APAs. We obtained the best results using the SD of the bias multiplied by a factor of 4 to determine the end of the APAs, and by filtering the signals at 3 Hz.

The resulting measures were compared with those from a stabilometer as a gold standard. The results suggested the usefulness of inertial sensors for detection and evaluation of APAs prior to stepping.

Angular velocity sensors are proposed to improve the detection of APAs, specifically, the end of APAs.

These results were obtained using a very simple algorithm. This algorithm is not computationally demanding and is simple enough to implement in low computational power devices such as digital signal processors (DSP), digital signal controllers (DSC), or even in microcontrollers, thus avoiding the use of expensive computers and improving the power of inertial sensors as a tool to evaluate APAs in an inexpensive way. Although the detection and waveform performance were not better than those of the stabilometer, they were sufficiently similar to provide a general idea of the status of the APA generation system. These inertial sensors could be used as a first-line tool for the diagnosis of APAs before stepping. It should also be noted that a better algorithm and improved signal processing, while probably more computationally demanding, could improve the overall results of the inertial sensors.

The development of a portable and reliable device to evaluate gait initiation in environments different from that of laboratories or hospitals could help in encouraging the participation of the entire population in the prevention of illness or early prediction of diseases, thereby achieving pervasive and personalized healthcare [25].

The results obtained in this study also increase the body of literature outlining gait initiation analysis using inertial sensors and reaffirm the results obtained by other researchers with respect to the duration and amplitude of APAs in healthy subjects. It must be noted that the algorithm was proved on healthy and young subjects and show similar results to those published previously in the same type of subjects [27]. However, further research must be done in elderly and patients where the baseline may be not so straight making difficult to detect the beginning of the APAs.

## Competing interests

The authors declare that they have no competing interests.

## Authors' contributions

RM was responsible for the design of the experiments, with important contributions from TT. MS designed and developed the hardware and software used in this experiment (except those clearly specified in the text), and the acquisition of data was carried out by RM. This document was drafted by RM, with important and substantial contributions from TT and MS, who also participated in data analysis. All of the authors have read and approved the final manuscript.
